# Call for manuscripts: "Towards a scaling-up of training and education for health workers"

**DOI:** 10.1186/1478-4491-5-14

**Published:** 2007-06-06

**Authors:** Mario R Dal Poz, Hugo Mercer, Margaret Gadon, Daniel MP Shaw

**Affiliations:** 1 Department of Human Resources for Health, Health Systems and Services, World Health Organization, 20 Avenue Appia, Geneva, Switzerland; 2 American Medical Association, 515 N. State Street, Chicago, IL 60610, USA

## Joint call for papers for special issue of the journals

***Education for Health ***()

***Human Resources for Health ***()

WHO and the journal *Education for Health *and *Human Resources for Health *are now accepting manuscripts for joint special issues addressing the critical need for a skilled, sustainable health workforce in the developing world. Submitted articles must fall under the broad theme:

"Towards a scaling-up of training and education for health workers"

The World Health Report 2006, *Working together for health*, recognized the centrality of the health workforce for the effective operation of country health systems and outlined proposals to tackle a global shortage of 4.3 million health workers. There is increasing evidence that that this shortage is interfering with efforts to achieve international development goals, including those contained in the Millennium Declaration and those of WHO's priority programmes.

The health workforce crisis in developing countries derives principally from inadequate educational opportunities for health workers and a lack of relevance of their training to community health care practice. Additional contributing factors include: inadequate compensation and working conditions, the deteriorating health of the workforce in many developing countries, urban/rural and workforce imbalance, and migration of the workforce from developing to developed countries.

### We are seeking manuscripts which concern the scaling-up of training and education for health workers. Possible sub-themes include, but are not limited to

• private sector engagement

• regulatory frameworks for education and practice

• labour market dynamics after the production of health workers (e.g. retention)

• training teams rather than individuals

• skills mix

• multi-skilled workers, responsive to exiting needs

• task-shifting/role substitution

• competency-based education and training

### Examples of questions that could be considered are

• What ongoing efforts to increase graduate level primary care training have been established in developing countries. What has been their impact and what have been their problems?

• What effective strategies have been developed and tested for customizing the workforce skill mix to local health service needs? For example, what impact have recent health sector reforms had on the local health workforce?

• What is the status of existing efforts to train health workers using innovative methods, including distance learning and various forms of information technology? How will training by protocol differ from, and complement, traditional community health worker training?

• How can the health professional training be better aligned with local health needs and be more socially accountable?

• What is the status of existing collaborations between developing countries aiming to improve health worker education?

• How have modifications in healthcare management had an impact upon health workforce capacity at the local level?

### Manuscripts will be accepted in two formats

Full papers of 3000 words or less for policy and research papers.

Brief communications of less than 1200 words: better suited to program or project descriptions or commentaries.

Planned publication is over the period from June to August 2008. There will be an online facility to respond to published articles in order to accommodate a live debate.

If you would like to submit either an article or brief, please send us a provisional title and a short outline of the major topics you would address.

**Proposals for manuscripts are due by 31 July 2007 and should be submitted by e-mail to **hrhspecial@who.int. **Instructions for submission of articles will then be provided with feedback. Final manuscripts are due by 30 October 2007.**

